# IgM Antibody Detection as a Diagnostic Marker for Acute Toxoplasmosis: Current Status of Studies and Main Limitations

**DOI:** 10.3390/antib14020044

**Published:** 2025-05-21

**Authors:** Karolina Sołowińska, Lucyna Holec-Gąsior

**Affiliations:** Department of Biotechnology and Microbiology, Faculty of Chemistry, Gdańsk University of Technology, 11/12 Narutowicza Str., 80-233 Gdańsk, Poland; karolina.solowinska@pg.edu.pl

**Keywords:** IgM, *Toxoplasma gondii*, diagnosis, toxoplasmosis, recombinant antigen

## Abstract

Accurate dating of *Toxoplasma gondii* infection is essential for effective clinical management, particularly in pregnant women and immunocompromised individuals, where distinguishing acute from chronic infection informs treatment decisions. Serological detection of IgM antibodies is a key tool in diagnosing recent toxoplasmosis; however, its reliability is compromised by persistent IgM responses, cross-reactivity, and assay variability. While IgM lacks sufficient specificity to serve as a standalone marker of acute infection, it remains an important component of serological panels. This review summarizes current IgM detection methods and explores advancements aimed at improving diagnostic accuracy with a focus on recombinant antigens, which have emerged as promising alternatives to traditional *Toxoplasma* lysate antigen-based immunoassays. This paper also explores alternative methods of differentiating chronic and acute toxoplasmosis and outlines key areas for future research.

## 1. Introduction

*Toxoplasma gondii*, an obligate intracellular protozoan parasite, remains a major global health concern owing to its wide distribution, broad range of hosts, and multiple transmission pathways [1].

The life cycle of *T. gondii* involves transitions between definitive and intermediate hosts, with distinct stages responsible for acute and chronic toxoplasmosis. The sexual phase occurs exclusively in the intestinal epithelium of felids, where oocysts are produced and shed in feces. After sporulating in the environment, these oocysts contaminate soil, water, and vegetation, enabling transmission to animals and humans [2]. Upon entering the intermediate host, *T. gondii* differentiates into tachyzoites, which disseminate through the bloodstream and lymphatic system, invading various tissues, leading to the symptomatic manifestations of acute infection [3]. Within a few days, tachyzoites transition into bradyzoites, forming tissue cysts—a characteristic feature of chronic *Toxoplasma* infection. Bradyzoites divide slowly within these cysts, which may eventually rupture, releasing parasites that initiate new rounds of infection and cyst formation [4]. This process allows the parasite to maintain a persistent infection within the host. In immunocompromised individuals, released bradyzoites can transform back into tachyzoites, leading to reactivation of infection and severe complications, such as toxoplasmic encephalitis [5]. Furthermore, tissue cysts serve as the infective stage for intermediate and definitive hosts, facilitating transmission through consumption of undercooked or raw meat [6].

Less frequently, infection may occur via blood transfusions, organ transplants from infected donors, drinking unpasteurized milk containing tachyzoites, or vertically through congenital transmission, which occurs when the parasite crosses the placental barrier during pregnancy [7].

The risk of fetal infection increases with gestational age due to greater placental permeability [8]. While first-trimester acute maternal infections have a low transmission rate (<10%), they are linked to the most severe outcomes. By the third trimester, transmission risk rises to 60–70%, though resulting disease is usually milder or subclinical [9]. Chronic toxoplasmosis in immunocompetent mothers poses minimal risk unless reactivation occurs; however, even then, transmission is rare [8].

Additionally, the virulence of *Toxoplasma* strains impacts clinical outcomes and geographic differences in strain distribution contribute to variations in disease severity worldwide [9]. *T. gondii* isolates can be categorized into three main clonal lineages (types I, II, and III), with type II prevalent in Europe and North America [10], as well as numerous atypical genotypes found more frequently in South America and Africa [11,12]. In congenital toxoplasmosis, infections caused by atypical *Toxoplasma* genotypes are associated with more severe clinical outcomes compared to those caused by typical type II strains [13].

Estimating the date of maternal infection informs several key aspects of care. First, distinguishing between infections acquired before conception and those occurring during pregnancy is crucial, as only the latter pose a risk for fetal transmission. Second, the timing of the infection relative to gestational age guides clinical decisions regarding treatment, the need for amniocentesis [14], the likelihood of fetal transmission, and the potential severity of the disease. Finally, amniocentesis is recommended to be performed at least one month after the estimated date of infection [15].

Serological tests are commonly employed to assess both immunoglobulin G (IgG) and immunoglobulin M (IgM) against *T. gondii*, providing an indication of past exposure and potential recent infection, respectively [2]. Despite the clinical utility of IgM detection for identifying acute toxoplasmosis, interpreting results can be challenging due to high false-positive rates [16]. This issue arises from cross-reactivity with other infections [17], the persistence of IgM antibodies long after the primary infection [18,19], and the presence of natural IgM that reacts with *Toxoplasma* antigens in the absence of actual infection [20,21]. As a result, IgM-based tests may lead to misdiagnoses, causing unnecessary patient anxiety, inappropriate clinical decisions, and potentially unwarranted medical interventions such as the decision to terminate the pregnancy [22].

This article provides an overview of the current state of research on the detection of *T. gondii*-specific IgM antibodies as a diagnostic marker of acute infection. It integrates both established knowledge and recent advancements in diagnostic methodologies, aiming to serve as a thorough and informative resource on IgM detection in the diagnosis of toxoplasmosis. The review outlines the various types of IgM immunoassays, including both automated and manual formats, and evaluates the use of recombinant antigens for their reactivity with anti-*T. gondii* antibodies. Particular emphasis is placed on the challenges associated with directly comparing findings across studies due to methodological variability and differences in study design. Finally, the article highlights alternative approaches for identifying recent infections and proposes future research directions to improve diagnostic accuracy and standardization in the field.

## 2. Kinetics of Antibody Response During *T. gondii* Infection

**Figure 1:**
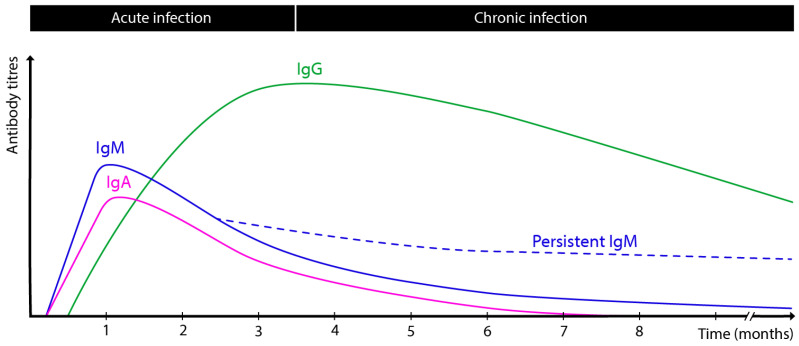
Kinetics of antibody response following *T. gondii* infection.

Serodiagnosis primarily relies on measuring IgM, IgG, and immunoglobulin A (IgA) antibody levels, each with distinct temporal profiles (Figure 1). Understanding these kinetics is vital for interpreting serological tests accurately and distinguishing acute from chronic *T. gondii* infection. IgM typically emerges about one week post-infection and peaks around one month, followed by a gradual decline over approximately seven months [2]. However, it is well known that 9–27% of patients exhibit persistent IgM for two years or longer, complicating the differentiation between recent and past infection [23].

The IgA response largely parallels that of IgM, though it peaks slightly later and wanes between four and nine months post-infection [24]. In contrast, IgG usually appears about two weeks after infection onset, peaks around three months, and remains at a plateau for roughly six months before slowly decreasing; nonetheless, low-level IgG can persist indefinitely, reflecting latent cyst presence [23].

The presence of IgM in sera is not unequivocally indicative of acute toxoplasmosis; however, IgM negativity can confidently exclude recent infection [25]. Comprehensive diagnostic panels—including IgG levels and IgG avidity—are essential for a more accurate clinical picture [26]. Typically, low-avidity IgG combined with IgM positivity supports a recent infection, whereas high-avidity IgG implies a longer-standing immune response [25]. It is important to note that pregnant women treated with spiramycin experience significantly delayed avidity maturation [27].

In practice, some laboratories may include additional markers, such as IgA testing [28]. Theoretically, IgA could serve as a valuable marker of recent infection due to its kinetic profile—characterized by a more rapid decline than IgM—and the absence of natural IgA in uninfected individuals. However, its utility in the prenatal and neonatal diagnosis of acute toxoplasmosis has been a subject of ongoing debate. The most recent study conducted on a large cohort concluded that while a negative IgA enzyme-linked immunosorbent assay (ELISA) result reduces the likelihood of a recent maternal infection, it does not definitively rule it out [29]. The inclusion of IgA testing was recommended for pregnant women testing both IgG- and IgM-positive [29]. In the case of neonatal diagnosis, a large-scale study conducted in 2022 [30] demonstrated that incorporating *T. gondii* IgA ELISA into the serological panel for congenital toxoplasmosis did not significantly enhance the overall diagnostic performance compared to IgM-based detection alone [30]. However, this assessment did not consider the influence of maternal treatment on the sensitivity of serological markers at birth. In a separate study, Guegan et al. [31] reported that IgM was detected less frequently in neonates born to treated mothers compared to those born to untreated mothers, whereas IgA detection remained unaffected by maternal therapy [31]. IgA testing may therefore offer particular diagnostic value in neonates exposed to prenatal anti-*Toxoplasma* treatment.

Although IgM is not a definitive, standalone marker of acute infection, it remains a crucial component within a multifaceted diagnostic approach. Consequently, ongoing efforts focus on enhancing IgM assay technologies to improve specificity, reduce false positives, and integrate more effectively with other serological markers to achieve a more accurate evaluation of patient status. Continued enhancement of IgM immunoassays is also crucial for diagnosing congenital toxoplasmosis because neonatal IgM is a key indicator of recent fetal infection. In contrast, IgG readily crosses the placenta and can remain in the infant’s bloodstream for 6–12 months, making it an unreliable marker of active congenital infection [9]. Interestingly, Cañedo-Solares et al. [32] identified a correlation between specific IgG subclasses in maternal sera and both the risk of congenital transmission and the severity of the disease [32].

Although identifying IgM in newborns is highly valuable, it should be interpreted cautiously, particularly with sensitive assays, as maternal IgM may be transmitted during childbirth [33]. A study analyzing a decade of postnatal congenital toxoplasmosis cases proposed recommendations for improving diagnostic practices [34]. The authors identified the compared immunological profile (CIP) assay—which involves simultaneous sampling of maternal and neonatal sera at birth to directly compare immunoglobulin levels—as the most effective diagnostic method, contributing to 98% of confirmed cases within the first 10 days of life and serving as the only positive marker in 19% of cases. Beyond day 10, continued diagnostic evaluation should include IgM and IgA tests in combination with CIP assays and regular monitoring of IgG levels [34].

## 3. Current Methods for IgM Antibody Detection

A variety of serological assays, differing in format and detection techniques, have been developed to detect IgM antibodies against *T. gondii*. Automated immunoassays generally offer high analytical sensitivity and specificity. Overall, the negative predictive value (NPV) of these platforms is uniformly high—meaning that a negative IgM result from any automated test essentially rules out a recent infection in immunocompetent patients. However, their positive predictive value (PPV) remains variable [35,36,37,38,39,40,41,42,43]. Furthermore, the specificity of IgM detection has been shown to vary between different production lots, even when sourced from the same manufacturer [44]. The most commonly used automated immunoassays (both currently available and discontinued) and their underlying principles are summarized in Table 1.

In practice, clinical laboratories usually employ ELISA or chemiluminescence immunoassay (CLIA) commercial kits and automated platforms as initial screening techniques, followed by confirmatory testing of IgM-positive or equivocal cases using follow-up samples or alternative methods [53]. Confirmatory testing is performed by reference laboratories and consists of more complex or expensive methods such as indirect fluorescent antibody test (IFAT), Western blot, and immunosorbent agglutination assay (ISAGA) [23].

IFAT is a microscopy-based method in which fixed tachyzoites on a slide serve as the antigen substrate. Diluted patient serum is applied, and if *Toxoplasma*-specific antibodies are present, they bind to the parasites and are visualized by a fluorescent anti-μ chain antibody [54]. Several difficulties have relegated IFAT to a secondary role. Reading the fluorescence is subjective and requires a trained eye, leading to inter-observer variability. Low-level IgM can be difficult to discern, and background fluorescence or cell debris can cause confusion. Moreover, false-positive IFAT IgM results can occur in the presence of rheumatoid factor or antinuclear antibodies, and high concentrations of IgG in a sample may competitively inhibit IgM binding, yielding false negatives [55]. Nevertheless, IgM IFAT can support the diagnosis of acute toxoplasmosis. A 2016 study reported that this method correctly identified a majority of recently infected pregnant women with a high positive predictive value (~85%) [56]. The authors concluded that IgM IFAT demonstrates adequate sensitivity and specificity for detecting recent infections and can serve as a supplementary tool for accurately diagnosing acute toxoplasmosis in pregnancy, particularly in cases with borderline avidity test results [56].

The ISAGA is an immunocapture assay, regarded as the gold standard for detection of *Toxoplasma* IgM in cases of congenital infection [57]. The method utilizes live tachyzoites as its primary antigen source, with TG-180 sarcoma cells incorporated to enhance tachyzoite production. Maintaining these cultures is a complex and labor-intensive process. Additionally, the test necessitates overnight incubation, and its interpretation relies on an experienced reader for accurate results [58]. ISAGA was commercialized by bioMérieux; however, due to changes in European regulations, the kits were discontinued in 2024, necessitating the development of alternative methods with comparable diagnostic accuracy [59]. In recent comparative studies, the automated Bio-Rad Platelia Toxo IgM has shown close equivalence to the ISAGA in detecting congenital toxoplasmosis [59,60].

Western blot (or immunoblot) is a valuable tool that complements conventional serological assays in the diagnosis of *Toxoplasma* infection. The technique detects serum antibodies that bind to *T. gondii* antigens, which are separated by electrophoresis and transferred onto a membrane, resulting in banding patterns that correspond to specific molecular weights [55]. It is particularly effective for early postnatal diagnosis of congenital toxoplasmosis, enabling qualitative comparison of IgG and IgM antibody profiles between maternal and neonatal sera. A commercial version of this method, the Toxoplasma Western Blot IgG/IgM kit (LDBio Diagnostics, Lyon, France), has demonstrated high diagnostic utility, reportedly offering superior sensitivity for IgM detection, outperforming the ISAGA technique [61]. An IgM-specific assay, the Toxo II IgM Western Blot (LDBio Diagnostics) has recently become commercially available. The test utilizes strips with five recombinant proteins (P30, P31, P33, P38 and P40). The presence of a minimum of two bands and the inclusion of the band P30 kDa allow the assay to be interpreted as positive. Meroni et al. [62] reported the tests clinical value in managing ambiguous serological profiles during pregnancy. The authors found that the test successfully identified nearly all seroconversions with a sensitivity of 97.8% and effectively distinguished false-positive results, demonstrating a specificity of 89.7% in samples that tested falsely positive with standard screening assays [62]. In contrast, a similar recombinant antigen-based immunoblot—the recomLine Toxoplasma IgM (Mikrogen GmbH, Neuried, Germany), which utilized eight recombinant antigens—was discontinued. This decision could be linked to poor performance, as a 2021 study [63] reported a sensitivity of only 39.3% in differentiating specific anti-*Toxoplasma* antibodies from natural IgM [63].

Lateral flow assays (LFAs), also known as immunochromatographic tests (ICTs), are a widely accepted point-of-care (POC) diagnostic approach [64]. Their simplicity and affordability make them especially valuable in resource-limited settings, where access to automated immunoassays and reference laboratories is often restricted due to high costs, the need for specialized equipment, and trained personnel [65]. Additionally, it serves as a practical tool for early pregnancy screening. These assays operate on the principle of capillary action, where a liquid sample—typically serum or whole blood—is driven along a nitrocellulose membrane, producing a visible result within 30 min [66]. Am evaluation of three commercially available *Toxoplasma* POC tests—the Toxo IgG/IgM Rapid Test (Biopanda, Belfast, UK), OnSite Toxo IgG/IgM Combo Rapid Test (CTK Biotech, Poway, CA, USA), and Toxoplasma ICT IgG-IgM-bk (LDBio Diagnostics, Lyon, France)—revealed marked differences in diagnostic performance [67]. The Biopanda and OnSite assays, which utilize recombinant *T. gondii* antigens and provide separate test bands for IgG and IgM, showed limited sensitivity for IgM detection (62.2% and 28%, respectively). In contrast, the LDBio test, which uses whole-cell tachyzoite lysates from the *T. gondii* RH Sabin Type I strain and detects IgG and IgM together in a single test band, achieved 100% sensitivity for combined IgG/IgM detection [67]. However, due to the combined detection format, it is not possible to confirm whether this sensitivity reflects true IgM detection. This limitation is particularly relevant given that another study reported that samples with isolated IgM positivity (IgM+/IgG−) were negative with the LDBio test [68]. This suggests that while the LDBio assay performs well in detecting combined IgG/IgM responses, its ability to detect IgM in isolation remains uncertain. In resource-limited settings, however, accessible and affordable POC tests may be the only viable diagnostic option, particularly in remote or underserved areas. Although their sensitivity may be lower than that of laboratory-based assays, LFAs offer a practical solution for initial screening, helping to identify individuals who may require further evaluation. In such contexts, even moderate diagnostic performance can be clinically valuable by enabling access to care where no other diagnostic infrastructure exists. Nevertheless, there remains a clear need for further advancements to enhance the sensitivity, specificity, and interpretive power of these tests, particularly in distinguishing between acute and chronic *Toxoplasma* infections—or the development of new POC diagnostics capable of addressing these limitations.

Overall, the detection of anti-*T. gondii* IgM involves a two-step approach, where automated immunoassays serve as the first-line screening tools, followed by more specialized confirmatory techniques to enhance diagnostic accuracy. Automated immunoassays are generally favored for first-line screening due to their efficiency, scalability, and high degree of standardization. These assays allow for high-throughput processing of large sample volumes with minimal manual input—an essential advantage in routine clinical settings. Their automated nature reduces the risk of human error and provides consistent, easily interpretable results. In contrast, alternative IgM detection methods (e.g., ISAGA, IFAT, or Western blot) are better suited for confirmatory testing. These techniques, while more accurate in certain contexts, are time-consuming, labor-intensive, and more expensive per sample. Moreover, their interpretation often requires expert evaluation, introducing a degree of subjectivity into the diagnostic process.

## 4. Recombinant Antigens in IgM Detection

Whole-tachyzoite antigens (TLAs) are commonly used in serological tests for *Toxoplasma* infection, and while they are effective in eliciting broad immune responses, they are inherently variable. The undefined antigenic composition of each batch, coupled with differences in parasite culture methods, leads to inconsistencies in diagnostic test performance across laboratories worldwide [69,70]. Reliance on crude parasite antigens not only introduces challenges in standardization and scalability but also may be biohazardous as handling live tachyzoites during production poses a risk to laboratory personnel and increases regulatory burdens [71]. Recombinant antigens offer a scalable, reproducible, and safer alternative, enabling the production of well-defined, high-purity proteins at lower costs [72]. The potential to replace native antigens with recombinant proteins in serodiagnosis of toxoplasmosis was first explored by Tenter and Johnson in 1991 [73]. Since then, numerous studies have confirmed the effectiveness of recombinant proteins in detecting anti-*T. gondii* antibodies [69,74,75,76]. Among these, surface antigens (SAGs), matrix antigens (MAGs), micronemal proteins (MICs), rhoptry proteins (ROPs), and dense granule antigens (GRAs) have been of particular interest.

Although TLAs can detect the presence of *T. gondii*-specific IgM antibodies with almost 100% sensitivity, their antigenic complexity and variability results in significant overlap in antibody detection during different stages of infection. This limitation reduces the reliability of TLA-based assays for accurately differentiating acute toxoplasmosis from chronic or past infections. In contrast, recombinant proteins offer a more precise diagnostic alternative by focusing on antigens uniquely expressed or highly immunogenic during acute toxoplasmosis. Moreover, chimeric and multi-epitope antigens, which combine immunodominant epitopes from multiple proteins, further enhance diagnostic sensitivity and specificity.

In order to identify stage specific antigens, studies report Western blot analyses using whole-tachyzoite antigens to find proteins recognized by anti-*T. gondii* IgM antibodies [77,78,79,80,81,82,83]. Additionally, transcriptomic studies have been conducted to identify mRNA transcripts uniquely expressed in specific developmental stages of the parasite [84,85]. Several recombinant proteins, both single (Table 2) and chimeric (Table 3), have been examined for their ability to react with *T. gondii*-specific IgM in human sera. The types of tested sera and classification criteria are shown in Table S1: Classification of serum samples tested in IgM ELISA based on individual recombinant *T. gondii* antigens and Table S2: Classification of serum samples tested in IgM ELISA based on chimeric recombinant *T. gondii* antigens, while the search strategy is described in Supplementary File S1: Search strategy.

Initial studies examined surface antigens of *T. gondii*, particularly SAG1 (P30) and SAG2 (P22) due to their well-known strong immunogenicity and stage-specific expression [105]. SAG2 has been shown to distinguish between acute and chronic IgG responses [106]. However, its application in anti-*Toxoplasma* IgM detection has proven limited. Parmley et al. [89] reported that only 46% of acute infection sera tested IgM-positive using a SAG2-based ELISA, concluding that this antigen is unsuitable for distinguishing recent from past infections through IgM immunoassays—a finding later corroborated by other studies [86,90]. Similarly, SAG1 exhibited low sensitivity in IgM detection, with reported values of 10.6% [86] and 39.3% [88]. Notably, the diagnostic performance of SAG1 improved significantly when applied in a double-sandwich ELISA format [87], which is to be expected, as it offers enhanced sensitivity and specificity by effectively detecting low antigen concentrations and reducing background interference [107]. However, this method introduces higher costs and procedural complexity. Additionally, the sensitivity of indirect ELISA may be impacted by the presence of both IgG and IgM in acute-phase sera which can lead to competitive binding to the antigen. Many other tested antigens lack comparative studies evaluating their performance in both indirect and sandwich formats; moreover, there is a notable lack of research assessing antigen utility across alternative immunoassay platforms beyond ELISA. This limits the understanding of antigen utility in different diagnostic contexts and may hinder the development of novel diagnostic tools.

Interestingly, findings by Teimouri et al. [71] contrast with earlier reports. In their study, SAG1-based IgM ELISA demonstrated high sensitivity (89.7%), specificity (96.3%), and strong agreement with commercial assays. This discrepancy highlights the lack of reproducibility across studies. The difficulty in comparing results across investigations may stem from the use of distinct serum panels, with geographic variation in *T. gondii* strain distribution likely contributing to the observed inconsistencies. Differences in recombinant protein production methods can also alter antigen conformation and immunoreactivity. Furthermore, inconsistencies in assay design, including antigen coating concentrations, detection systems, and cut-off values, introduce additional layers of variability. Taken together, these factors underscore the difficulty in comparative analysis of immunodiagnostic performance of recombinant proteins.

Among dense granule antigens, GRA8 (P35) has been most extensively studied for its potential as a diagnostic marker for recent *Toxoplasma* infection. Aubert et al. [86] investigated whether detecting IgM antibodies to P35 (amino acid residues 1–135 fused to the *Escherichia coli* CKS protein) could serve as an accurate marker for acute infection in pregnant women. Their study reported a 46.1% sensitivity for detecting IgM in sera classified as acute using Abbott IMx Toxo IgM and IgG assays [86]. In the same year, Suzuki et al. [93] employed a double-sandwich ELISA using GST-labeled P35_1–135_, achieving 90% sensitivity and 100% specificity in distinguishing acute sera (high dye test (DT) titers, positive IgM and IgA ELISA, and acute patterns in the AC/HS test) from chronic sera. The same article reported that 12 of 16 sera with persistent IgM antibodies (low DT titers and negative IgA) lacked detectable IgM specific to GRA8, highlighting its specificity for acute infection [93]. Differences in assay sensitivity between the described studies can again be attributed to variations in ELISA procedures as described previously.

Suzuki et al. [93] also tested sera from four women who seroconverted during pregnancy. Both the P35-IgM-ELISA and conventional IgM ELISA detected antibodies in all samples within two months of seroconversion. However, by 4–6 months, P35-IgM-ELISA was positive in only two samples, whereas conventional ELISA remained positive for all [93]. This suggests that IgM titers detected by ELISA based on P35 decline more rapidly after infection compared to conventional methods. Further studies supported these findings. Lu et al. [94] reported that sequential testing of P35-specific antibodies in patients revealed that IgM titers declined sharply after the acute phase, with positive rates dropping to 33% by the fifth month after seroconversion [94]. This indicates that P35-IgM is a specific marker for acute infection, outperforming assays that rely on whole-tachyzoite antigens. Moreover, the study confirmed previous findings, that most patients with persistent IgM antibody were P35-IgM negative [94]. The evaluation of new recombinant antigens for IgM detection must include testing with sera from individuals exhibiting persistent IgM responses. Despite its importance, many studies omit testing with persistent IgM-positive sera, focusing instead solely on acute, chronic, and negative samples. This oversight may lead to an overestimation of assay specificity and limit clinical utility.

Subsequent studies have explored different fragments of the GRA8 antigen. Babaie et al. [95] reported the production of a fragment spanning amino acids 23–169. While this antigen was highly specific, with 97.1% of chronic sera testing negative, its sensitivity was limited to 60.6% [95]. Later, Costa et al. [96] expressed GRA8_1–95_ and GRA8_48–145_, demonstrating that indirect ELISA using these fragments was ineffective for distinguishing acute from chronic infection; however, the IgG avidity assay showed high diagnostic accuracy, with 85.71% sensitivity and 100% specificity [96]. This suggests GRA8 may also be valuable for IgG avidity assays. The presented findings highlight the importance of selecting appropriate amino acid fragments of antigens for immunoassays. Full-length proteins may contain regions that are poorly expressed, non-immunogenic, or that contribute to cross-reactivity, potentially compromising assay specificity and sensitivity. In contrast, selecting immunodominant, epitope-rich fragments, particularly those that are conserved across pathogenic strains, can enhance antigen stability, expression yield, and immunoreactivity. These fragments can be identified through experimental methods, such as epitope mapping [108], or through bioinformatic analyses that predict B-cell epitopes and structural features associated with antigenicity [109]. Moreover, fragment selection allows for the exclusion of structurally disordered or post-translationally modified regions, which may not be accurately reproduced in heterologous expression systems. As such, rational design and selection of antigenic protein fragments play a key role in optimizing recombinant antigens for serological diagnostics.

Chimeric proteins are reported to offer significant advantages in the serodiagnosis of toxoplasmosis by improving sensitivity, specificity, and overall diagnostic accuracy [110]. By combining immunodominant epitopes from multiple antigens, these proteins enhance antibody recognition. Drapała et al. [103] constructed a chimeric P35-MIC1 protein which demonstrated 81.8% reactivity with sera from acute toxoplasmosis cases and 6.6% reactivity with chronic-phase sera in an indirect IgM-ELISA [103]. The reported sensitivity was significantly higher in comparison to single GRA8 tested in the same type of immunoassay. Interestingly, Ferra et al. [104] revealed that both the specific amino acid fragments and the arrangement these fragments within chimeric proteins influence diagnostic accuracy [104].

Buffolano et al. [87] described the use of recombinant antigens for the diagnosis of congenital toxoplasmosis in newborns. The authors found that an IgM ELISA based on a mixture of MIC2, MIC3, and SAG1 proteins correctly identified infection in 97% of infants as soon as 2 months after birth, compared to 29% detected by the whole-cell immunoassays [87]. Similarly, Beghetto et al. [100] reported enhanced diagnostic performance using chimeric recombinant antigens composed of immunodominant regions from MIC2, MIC3, and SAG1 or from GRA3, GRA7, and M2AP. Using these chimeric constructs in an IgM ELISA, 70% of infants with congenital toxoplasmosis tested positive, compared to only 35% detected by conventional whole-cell antigen-based assays [100]. However, it is important to note that the maternal treatment status was not accounted for in the study despite its known influence on the immunoglobulin response in neonates, which could significantly impact IgM detectability and overall assay sensitivity.

Virus-like particles (VLPs) have been explored as a platform to enhance *T. gondii* antigen presentation for improved immunogenicity and serological detection. Using the baculovirus expression system, researchers generated influenza matrix protein (M1) VLPs of a spherical morphology, with ROP4 forming spike-like projections on the surface [111]. This structural similarity to native virions enhances antigen presentation, potentially improving immune recognition. The diagnostic potential of ROP4 VLPs was evaluated in mice infected with *T. gondii* [112]. Results demonstrated significantly higher sensitivity in detecting IgG, IgM, and IgA antibodies compared to conventional TLA-based assays. Notably, ROP4 VLPs successfully detected all immunoglobulin classes as early as one-week post-infection, suggesting their utility in early-stage diagnosis. However, further validation using well-defined human serum samples is necessary to confirm these findings. Additionally, longitudinal studies assessing the kinetics of ROP4 VLP-IgM titers would be essential to determine their potential for differentiating acute from chronic *T. gondii* infections. Kim et al. [113] explored AMA1 VLPs, which exhibited strong reactivity with sera from mice infected with both RH and ME49 *T. gondii* strains. Compared to TLA, AMA1 VLPs elicited significantly higher IgG and IgM responses, demonstrating enhanced immunoreactivity. However, a prolonged IgM response complicated differentiation between acute and early chronic infections, limiting their specificity for recent infections [113]. This suggests that while recombinant antigens on the surface of VLPs outperform conventional TLA in overall sensitivity, their inability to produce a transient IgM response makes them less suitable as a definitive marker for acute toxoplasmosis.

## 5. Discussion

The presence of *Toxoplasma*-specific IgM antibodies is a key serological marker of recent infection, typically appearing within a week of exposure [55]. However, IgM persistence can extend beyond a year, making it difficult to differentiate newly acquired infections from past exposure [19]. Additionally, cross-reactivity, nonspecific immune responses, and assay variability further complicate interpretation [21]. As a result, a single IgM-positive result lacks standalone diagnostic value and must be interpreted alongside additional serological markers, such as IgG, IgA, or IgG avidity [26].

IgM testing is especially important for pregnant women where distinguishing acute from chronic infection guides management [114]. Although seroconversion remains the most reliable marker of recent infection, the implementation of routine maternal screening programs remains a topic of debate. Countries like France have conducted prenatal screening for several decades, while Brazil has only recently introduced nationwide neonatal screening in response to its high disease prevalence. In contrast, some European countries, including Spain, Switzerland, and the UK, have discontinued their programs. The ongoing debate primarily revolves around the cost-effectiveness of screening, as well as the lack of conclusive evidence that early treatment significantly reduces transmission [115,116].

Point-of-care serological tests, such as ICTs, may provide a cost-effective and accessible alternative, particularly in resource-limited settings [65]. Their ease of use and rapid turnaround make them appealing for widespread application, especially in regions with limited healthcare infrastructure. However, currently available ICTs face significant limitations. Many lack the sensitivity required for reliable detection of *T. gondii* infection or are unable to distinguish between IgM and IgG antibodies. This inability to differentiate acute from chronic infections presents a critical gap in the effective diagnosis and management of toxoplasmosis. The development of accurate, rapid, and affordable POC tests is especially urgent in regions such as South America and Africa, where more virulent *T. gondii* strains are prevalent and contribute to more severe forms of congenital toxoplasmosis [117]. Addressing this diagnostic shortfall is essential to improving early detection, timely treatment, and overall disease outcomes in high-risk populations.

As atypical *T. gondii* genotypes are linked to more aggressive disease presentations, there is a need to reassess current maternal testing protocols. Existing guidelines predominantly reflect epidemiological patterns observed in regions where type II strains are most common. As a result, infections with more virulent atypical strains may go unrecognized in clinical practice despite their association with more severe outcomes in congenital toxoplasmosis.

In clinical practice, IgM detection follows a two-step approach, beginning with automated immunoassays such as ELISA and CLIA, which serve as primary screening tools due to their high-throughput capacity and strong negative predictive value (NPV). While these methods effectively rule out infection in seronegative patients, their positive predictive value (PPV) varies, necessitating confirmatory testing with assays such as the ISAGA, IFAT, and Western blot. Historically, the ISAGA was regarded as the gold standard for IgM detection, particularly in congenital toxoplasmosis, but changes in European regulations led to its commercial discontinuation in 2024 [58]. The Bio-Rad Platelia Toxo IgM assay has since emerged as a viable alternative, demonstrating comparable diagnostic accuracy [59,60].

Despite the availability of multiple diagnostic approaches, the search for novel markers of acute toxoplasmosis remains an active area of research. Recombinant antigens have gained attention as a viable replacement for traditional TLA-based immunoassays. These antigens offer easier standardization. Further enhancing sensitivity and specificity, chimeric and multi-epitope antigens combine immunodominant fragments from multiple proteins, leading to greater overall diagnostic accuracy [69]. Both the selection of appropriate amino acid fragments and their arrangement within chimeric proteins significantly influence diagnostic performance, highlighting the need for further research to optimize recombinant antigen-based serodiagnosis of toxoplasmosis.

Comparative analysis of recombinant antigen performance is challenging due to variations in experimental methodologies. Differences in ELISA protocols, including assay format, antigen coating concentrations, detection systems, and cut-off values, contribute to variability between studies. Moreover, inconsistencies in statistical analysis further complicate direct comparisons. Differences is recombinant antigen production, such as expression system, fusion tags, and purification methods further increase the differences between studies. A critical limitation lies in the use of distinct serum panels across studies. Criteria for defining acute and chronic infection vary widely, ranging from the presence or absence of IgM to more comprehensive approaches involving IgG avidity testing or multiple serological markers. Many studies fail to assess whether recombinant antigens can reliably distinguish acute infection from persistent IgM, limiting their diagnostic validation. The global variation in parasitic genotypes further contributes to these challenges, as certain strains elicit stronger immune responses than others [117], affecting the generalizability of findings. Given the combined impact of technical, methodological, and biological factors, interpretation of recombinant antigen performance must be approached with caution, and standardized evaluation protocols are needed to support the development of reliable serodiagnostic tools.

A novel approach to diagnosing acute toxoplasmosis involves measuring YKL-40 levels, a glycoprotein also known as chitinase-3-like protein 1 (CHI3L1). This molecule, part of the chitinase-like protein family, has been explored as a biomarker for inflammatory and infectious diseases [118]. Shahad et al. [119] investigated whether YKL-40 levels are elevated in toxoplasmosis and whether they differ between the acute and chronic phases of the infection. Statistical analysis revealed highly significant differences in YKL-40 levels between toxoplasmosis patients and healthy controls, as well as between acute and chronic infection groups, with the highest levels observed in acute infections. However, further research is needed, as no additional studies have yet confirmed these findings. Other molecules investigated as potential biomarkers for recent *T. gondii* infection include microRNAs (miRNAs), though research in this area remains limited. MiRNAs are small, non-coding RNAs that regulate gene expression by either degrading mRNA or inhibiting its translation. Found in serum, plasma, urine, and other body fluids, they are gaining attention as non-invasive tools for disease diagnosis, monitoring, and prognosis [120]. Studies show that *T. gondii* infection alters miRNA expression in the mouse brain and spleen [121,122]. This presents a promising avenue for future research, as miRNAs may be detectable in peripheral blood before IgM antibodies appear or may be more specific to acute infection, potentially reducing false-positive results and addressing the issue of persistent IgM.

CRISPR (Clustered Regularly Interspaced Short Palindromic Repeats)-based diagnostic technologies have recently emerged as promising tools for nucleic acid detection, especially in low-resource settings and large-scale screening programs, due to their minimal equipment requirements and adaptability to lateral flow formats [123]. Techniques such as SHERLOCK (Specific High-sensitivity Enzymatic Reporter unLOCKing), which utilizes Cas13 for RNA detection, and DETECTR (DNA Endonuclease-Targeted CRISPR Trans Reporter), which employs Cas12 for DNA detection, offer highly sensitive, rapid, and portable platforms for the diagnosis of infectious diseases [124]. Despite their demonstrated utility in detecting a range of pathogens, CRISPR-based methods have not yet been evaluated for the detection of *T. gondii*, presenting an interesting prospect for future research.

Despite its limitations and challenges, IgM detection remains a fundamental tool in the diagnosis of acute toxoplasmosis. However, challenges identified decades ago remain relevant today, leading to the conclusion that, although IgM detection will likely continue to play an integral role in diagnostic panels, significant progress will ultimately depend on the exploration of alternative biomarkers of recent infection. The future of anti-*Toxoplasma* IgM testing should focus on developing inexpensive tools capable of accurately distinguishing acute infections from persistent IgM, designed for use in non-reference laboratories to increase accessibility and reliability across diverse clinical settings.

## Figures and Tables

**Table 1:** Automated immunoassays for detection of anti-*T. gondii* IgM.

Test	Manufacturer	Type of Test	Principle	Reference
Platelia Toxo IgM	Bio-Rad	EIA ^1^	Patient IgM antibodies bind to anti-human µ-chain antibodies coated on the microplate wells, followed by incubation with *T. gondii* antigen and a horseradish peroxidase (HRP)-conjugated murine monoclonal antibody anti-*T. gondii* (P30). Detection by chromogenic substrate (TMB).	[45]
Architect/Alinity	Abbott	CMIA ^2^	μ-Capture chemiluminescent immunoassay. Patient IgM antibodies bind to anti-human IgM-coated paramagnetic microparticles. Detection is achieved by incubating the bound IgM with native *T. gondii* lysate, pre-complexed with an acridinium-labeled anti-Toxo P30 (SAG1) monoclonal F(ab′)_2_ fragment. The resulting chemiluminescent signal is measured.	[37]
Advia Centaur/Atellica Toxo IgM	Siemens	CMIA	Patient IgM binds to mouse anti-human IgMμ monoclonal antibody covalently coupled to paramagnetic particles. A *T. gondii* antigen, complexed with an acridinium-labeled anti-p30 F(ab′)_2_ fragment, then binds to captured IgM. If IgM is present, antibody/antigen complexes form, producing a chemiluminescent signal proportional to IgM concentration.	[46]
Liaison	DiaSorin	CMIA	Magnetic particles coated with IgG to human IgM (mouse monoclonal) selectively bind IgM from the patient sample. After incubation with inactivated *T. gondii* (RH strain) obtained from ruptured and detergent-extracted trophozoite detection is achieved using mouse monoclonal anti- SAG1 antibodies conjugated to an isoluminol derivative.	[47]
Elecsys Toxo IgM	Roche Diagnostics GmbH	ECLIA ^3^	Patient sample is incubated with a ruthenium-labeled recombinant *T. gondii* SAG1 antigen, allowing IgM antibodies to bind. Then, biotinylated anti-human IgM antibodies and streptavidin-coated microparticles are added, forming a solid-phase complex via biotin/streptavidin interaction. After washing, the complex is held in an electrochemiluminescence measuring cell using a magnetic field, while unbound components are removed. An electrochemiluminescent reaction is triggered by applying voltage, and the light emission is detected.	[43]
VIDIA Toxo IgM	BioMérieux	CMIA	The system uses a two-step enzyme immunoassay on paramagnetic microparticles with a chemiluminescence-based detection step, but to the best of our knowledge, a complete description of the system has not been published.	[38]
AxSYM Toxo IgM	Abbott	MEIA ^4^	Microparticles are coated with *T. gondii* antigen (RH strain) derived from HeLa cell culture which captures IgM from serum. After washing, goat anti-human IgM–alkaline phosphatase conjugate binds to any attached IgM. A rheumatoid neutralization buffer reduces rheumatoid factor interference. The alkaline phosphatase reacts with the substrate 4-methylumbelliferyl phosphate (MUP) to release a fluorescent product.	[48]
Vidas Toxo IgM	BioMérieux	ELFA ^5^	Competitive fluorescent detection system, where patient IgM antibodies to *T. gondii* compete with an anti-p30 monoclonal antibodies. A Solid Phase Receptacle (SPR) serves as both the pipette and solid-phase support; its interior is precoated with *T. gondii* antigen (RH strain). Patient serum is drawn into the SPR from a reagent strip containing sequential wells for washing, conjugate incubation (alkaline phosphatase-labeled anti-p30 mAb), and fluorescent substrate (MUP). Any bound enzyme conjugate converts the substrate to a fluorescent product (4-methylumbelliferone).	[49]
BioPlex 2200 ToRC IgM kit	Bio-Rad	MFI ^6^	A multiplex system that simultaneously detects *T. gondii*, Rubella virus-, and CMV-specific IgM antibodies. Samples are incubated with antigen-coated fluoromagnetic beads carrying a unique fluorescent signature and binding IgM antibodies. Next, a fluorescent anti-human IgM reporter conjugate is added. As the bead mixture passes through a dual-laser flow detector, the first laser classifies each bead type by its embedded dye, while the second laser quantifies the fluorescence signal.	[50]
Immulite 2000	DPC-Siemens	CLIA	A polystyrene bead coated with partially purified *T. gondii* antigen (RH strain tachyzoites from mouse peritoneum) is incubated with patient serum. After washing, a goat anti-human IgM antibody conjugated to alkaline phosphatase is added, after which a chemiluminescent substrate (phosphate ester of adamantyl dioxetane) is introduced. To minimize false reactivity, reagents include antibodies to human IgG and rheumatoid factors.	[51]
Vitros	Ortho-Clinical Diagnostics	CLIA	Patient IgM is incubated with a biotinylated mouse anti-human IgM antibody, forming an immune complex that is captured by streptavidin on the test wells. Then, an HRP-labeled mouse monoclonal anti-*Toxoplasma* antibody—complexed with inactivated *T. gondii* antigen—binds to any *Toxoplasma*-specific IgM on the well. A luminogenic substrate and an electron transfer agent (a substituted acetanilide which increases the level of light produced and prolongs its emission).	[42]
Access	Beckman-Coulter	CMIA	Patient sample is added to paramagnetic microparticles coated with sheep anti-human IgM, allowing *T. gondii*-specific IgM to bind. A *T. gondii* antigen complexed with anti-p30 monoclonal antibody conjugated to alkaline phosphatase is introduced followed by addition of a chemiluminescent substrate.	[52]

^1^ EIA—enzyme immunoassay; ^2^ CMIA—chemiluminescent microparticle immunoassay; ^3^ ECLIA—electrochemiluminescence immunoassay; ^4^ MEIA—microparticle enzyme immunoassay; ^5^ ELFA—enzyme-linked fluorescent immunoassay; ^6^ MFI—multiplex flow immunoassay.

**Table 2:** Comparison of the diagnostic performance of individual recombinant *T. gondii* antigens in IgM ELISA using human serum samples.

Antigen Category	Antigen	Number of Tested Sera	Type of ELISA	Sensitivity (%)	Specificity (%)	Reference
Surface antigens (SAGs)	SAG1 (P30)	142	Indirect	10.6	ND *	[86]
	SAG1_45–196_	104	Double sandwich	65.7	95.8	[87]
	SAG1	58	Indirect	39.3	80	[88]
	SAG1	138	Indirect	89.7	96.3	[71]
	P22_27–172_ (SAG2)	26	Indirect	46	100	[89]
	SAG2	58	Indirect	64.3	83.3	[88]
	SAG2L_1–188_	242	Indirect	52.3	61.4	[90]
	SAG2c_27–173_			81.8	42.9	
	SAG3	58	Indirect	17.9	76.7	[88]
Dense granule antigens (GRAs)	GRA6_40–230_	88	Indirect	91.7	97.1	[91]
	GRA7 (P29)	142	Indirect	50.7	ND *	[86]
	GRA7	174	Indirect	96	90	[92]
	GRA7	138	Indirect	100.0	96.3	[71]
	GRA8_1–135_ (P35)	69	Double sandwich	90	100	[93]
	GRA8_1–135_ (P35)	142	Indirect	54.9	ND *	[86]
	GRA8 (P35)	125	Double sandwich	100	96	[94]
	GRA8_23–169_	68	Indirect	60.6	97.1	[95]
	GRA8A_1–95_	123	Indirect	57.8	59.3	[96]
	GRA8B_48–145_			65.2	59.3	
Rhoptry proteins (ROPs)	ROP1 (P66)	142	Indirect	58.5	ND *	[86]
	ROP2_196–561_	103	Indirect	62.1	100	[97]
	ROP2_177–537_ (P54)	142	Indirect	12.6	ND *	[86]
	ROP2_186–533_	203	Indirect	100	100	[98]
Micronemal proteins (MICs)	MIC2a_157–235_	104	Double sandwich	60	100	[87]
	MIC2b_466–610_			51.4	98.6	
	MIC3_234–307_			31.4	100	
Other	P68	142	Indirect	18.3	ND *	[86]
	M2AP_37–263_^1^	104	Double sandwich	48.6	98.6	[87]
	AMA1 ^2^	156	Indirect	80	93.8	[99]

* ND—No data; ^1^ M2AP—MIC2-associated protein; ^2^ AMA1—apical membrane antigen.

**Table 3:** Comparison of the diagnostic performance of individual chimeric *T. gondii* antigens in IgM ELISA using human serum samples.

Antigen	Number of Tested Sera	Type of ELISA	Sensitivity (%)	Specificity (%)	Reference
EC2 (MIC2_157–235_-MIC3_234–307_-SAG1_182–312_)	157	Double sandwich	98	100	[100]
EC3 (GRA3_36–134_-GRA7_24–102_-M2AP_37–263_)			84	100	
MAP1 ^1^ (SAG1-GRA7-GRA1)	250	Indirect	100	100	[101]
MEP ^2^ (SAG1_309–318_-SAG2_109–118_-SAG3_347–356_)	161	Indirect	96.6	100	[102]
P35-MAG1	123	Indirect	81.8	93.4	[103]
MIC1-ROP1			72.7	95.1	
MAG1-ROP1			59.1	96.7	
SAG2_31–170_-GRA1_26–190_-ROP1_85–396_-AMA1N_67–287_	207	Indirect	90.9	97.1	[104]
AMA1N_67–287_-SAG2_31–170_-GRA1_26–190_-ROP1_85–396_			84.9	99	
AMA1C_287–569_-SAG2_31–170_-GRA1_26–190_-ROP1_85–396_			92.4	91.4	
AMA1_68–569_-SAG2_31–170_-GRA1_26–190_-ROP1_85–396_			95.5	99	

^1^ MAP1—Multiple antigen peptide; ^2^ MEP—Multi-epitope peptide.

## Data Availability

No new data were created or analyzed in this study. Data sharing is not applicable to this article.

## Supplementary Materials

The following supporting information can be downloaded at: https://www.mdpi.com/article/10.3390/antib14020044/s1, File S1: Search strategy; Table S1: Classification of serum samples tested in IgM ELISA based on individual recombinant *T. gondii* antigens [71,83,86,87,88,89,90,91,92,94,95,96,97,98,99]; Table S2: Classification of serum samples tested in IgM ELISA based on chimeric recombinant *T. gondii* antigens [100,101,102,103,104].
