# Supplementary material for: IgM Antibody Detection as a Diagnostic Marker for Acute Toxoplasmosis: Current Status of Studies and Main Limitations
Source: Antibodies (Basel). 2025 May 21;14(2):44. doi: 10.3390/antib14020044 (PMC12101336; doi:10.3390/antib14020044)
Supplement: Supplementary file 1 [file antibodies-14-00044-s001.zip › File S1_supplementary file.pdf]

File S1. Search strategy

A literature search was conducted to identify studies evaluating recombinant proteins for the detection of anti-*T. gondii* IgM antibodies. The search was performed in the following electronic databases: PubMed, Scopus, and Google Scholar, using the following combination of keywords and Boolean operators: ("*Toxoplasma gondii*" OR "*T. gondii*" OR "toxoplasmosis") AND ("diagnosis" OR "recombinant protein") AND ("acute" OR "IgM"). In addition, manual searches of reference lists from the selected articles were performed to identify additional relevant studies. No publication date restrictions were applied.

Titles and abstracts were independently screened by both authors, and full texts of potentially eligible studies were reviewed.

Studies were included based on the following criteria: (1) published in English; (2) focused on the use of recombinant *T. gondii* proteins in IgM ELISA assays; and (3) utilized human serum samples in the immunoassays.

Studies not meeting the inclusion criteria were excluded. For each included study, data were extracted on the recombinant protein sequence used, the number and classification of serum samples tested, and the reported sensitivity and specificity of the assay.
