# Supplementary material for: IgM Antibody Detection as a Diagnostic Marker for Acute Toxoplasmosis: Current Status of Studies and Main Limitations
Source: Antibodies (Basel). 2025 May 21;14(2):44. doi: 10.3390/antib14020044 (PMC12101336; doi:10.3390/antib14020044)
Supplement: Supplementary file 1 [file antibodies-14-00044-s001.zip › Table S1_supplementary file.pdf]

Table S1. Classification of serum samples tested in IgM ELISA based on individual recombinant *T. gondii* antigens.

Antigen category	Antigen	Number of tested sera	Classification	Criteria	Reference
Surface antigens (SAG)	SAG1 (P30)	142	Acute infection sera (n=89)	IgG^+, IgM^+, IgA^+	[87]
			Recent seroconversion sera (n=53)	IgG^-, IgM^+, IgA^+	
	$SAG1_{45-196}$	104	Uninfected infant sera (n=69)	IgM^-	[88]
			Infected infant sera (n=35)	IgM^+	
	SAG1	58	Group 1 (n=30)	IgM^-	[89]
			Group 2 (n=28)	IgM^+	
	SAG1	138	Acute infection sera (n=29)	IgG^+, IgM^+, low IgG avidity	[72]
			Chronic infection sera (n=69)	IgG^+, IgM^-, high IgG avidity, no IgG increase or IgM detection in follow up samples.	
			Negative infection sera (n=40)	IgG^-, IgM^-	
	$P22_{27-172}$ (SAG2)	26	Acute infection sera (n=13)	High DT titers, IgM^+, IgA^+, acute patterns by the AC/HS test	[90]
			Chronic infection sera (n=13)	IgM^-, IgA^-, chronic patterns by the AC/HS test	
	SAG2	58	Group 1 (n=30)	IgM^-	[89]
			Group 2 (n=28)	IgM^+	
	$SAG2L_{1-188}$<br>$SAG2c_{27-173}$	242	Acute infection sera (n=85)	IgG^+, IgM^+, IgA^+, low IgG avidity	[91]
			Chronic infection sera (n=84)	IgG^+, IgM^-, high IgG avidity	
			Negative infection sera (n=73)	IgG^-, IgM^-	
	SAG3	58	Group 1 (n=30)	IgM^-	[89]
			Group 2 (n=28)	IgM^+	
Dense granule antigens (GRA)	$GRA6_{40-230}$	88	Acute infection sera (n=24)	IgM^+, IgG^+, seroconversion confirmed by two samples taken three weeks apart	[92]
			Chronic infection sera (n=34)	IgM^-, IgG^+	
			Negative infection sera (n=30)	IgM^-, IgG^-,	
	GRA7 (P29)	142	Acute infection sera (n=89)	IgG^+, IgM^+, IgA^+	[87]
			Recent seroconversion sera (n=53)	IgG^-, IgM^+, IgA^+	
	GRA7	174	Acute infection sera (n=70)	$IgG^{+/-}$, IgM^+	[93]
			Chronic infection sera (n=74)	IgG^+, IgM^-	
			Negative infection sera (n=30)	IgM^-, IgG^-	
	GRA7	138	Acute infection sera (n=29)	IgG^+, IgM^+, low IgG avidity	[72]
			Chronic infection sera (n=69)	IgG^+, IgM^-, high IgG avidity, no IgG increase or IgM detection in follow up samples.	
			Negative infection sera (n=40)	IgG^-, IgM^-	
	$GRA8_{1-135}$ (P35)	69	Acute infection sera (n=20)	High DT titers, IgM^+, IgA^+, acute patterns by the AC/HS test	[84]
			Chronic infection sera (n=33)	low DT titers, IgM^-, IgA^-, chronic patterns by the AC/HS test	
			Persisting IgM antibodies (n=16)	low DT titers, IgM^+, IgA^-, chronic patterns in the AC/HS test	
	$GRA8_{1-135}$ (P35)	142	Acute infection sera (n=89)	IgG^+, IgM^+, IgA^+	[87]
			Recent seroconversion sera (n=53)	IgG^-, IgM^+, IgA^+	
	GRA8 (P35)	125	Acute infection sera (n=25)	IgM^+, IgG^+, IHAT titer > 1:256. Seronegative date known	[95]
			Chronic infection sera (n=25)	IgM^-, IgG^+, IHAT titer < 1:64	

			Persisting IgM (n=25)	IgM^+, IgG^+, IHAT titer > 1:256	
			Recent seroconversion (n=25)	IgM^+, IgG^+, IHAT titer > 1:256	
			Negative infection sera (n=25)	IgM^-, IgG^-, IHAT titer < 1:64.	
	$GRA8_{23-169}$	68	Acute infection sera (n=33)	IgM^+, IgG^+, low IgG avidity	[96]
			Chronic infection sera (n=35)	IgM^-, IgG^+	
	$GRA8A_{1-95}$	123	Acute infection sera (n=45)	IgG^+, IgM^+, IgA^+, low IgG avidity	[97]
			Chronic infection sera (n=27)	IgG^+, IgM^-, high IgG avidity	
	$GRA8B_{48-145}$		Negative infection sera (n=51)	IgG^-, IgM^-	
Rhoptry proteins (ROP)	ROP1 (P66)	142	Acute infection sera (n=89)	IgG^+, IgM^+, IgA^+	[87]
			Recent seroconversion sera (n=53)	IgG^-, IgM^+, IgA^+	
	$ROP2_{196–561}$	103	Group 1 (n=35)	IgG^+, IgA^-, IgM^-	[98]
			Group 2 (n=21)	IgG^+, IgA^+, IgM^+	
			Group 3 (n=5)	IgG^+, IgA^+, IgM^-	
			Group 4 (n=16)	IgG^+, IgA^-, IgM^+	
			Group 5 (n=26)	IgG^-, IgA^-, IgM^-	
	$ROP2_{177-537}$ (P54)	142	Acute infection sera (n=89)	IgG^+, IgM^+, IgA^+	[87]
			Recent seroconversion sera (n=53)	IgG^-, IgM^+, IgA^+	
	$ROP2_{186-533}$	203	Acute infection sera (n=48)	IgG^-, IgM^+	[99]
			Chronic infection sera (n=59)	IgG^+, IgM^-	
			Negative infection sera (n=96)	IgM^-, IgG^-	
Micronemal proteins (MIC)	$MIC2a_{157-235}$	104	Uninfected infant sera (n=69)	IgM^-	[88]
	$MIC2b_{466-610}$		Infected infant sera (n=35)	IgM^+	
	$MIC3_{234-307}$				
Other	P68	142	Acute infection sera (n=89)	IgG^+, IgM^+, IgA^+	[87]
			Recent seroconversion sera (n=53)	IgG^-, IgM^+, IgA^+	
	$M2AP_{37-263}$	104	Uninfected infant sera (n=69)	IgM^-	[88]
			Infected infant sera (n=35)	IgM^+	
	AMA1[2]	156	Acute infection sera (n=40)	IgG^+, IgM^+, low IgG avidity	[100]
			Chronic infection sera (n=40)	IgG^+, IgM^-, high IgG avidity	
			Negative infection sera (n=76)	IgG^-, IgM^-	
