# Supplementary material for: IgM Antibody Detection as a Diagnostic Marker for Acute Toxoplasmosis: Current Status of Studies and Main Limitations
Source: Antibodies (Basel). 2025 May 21;14(2):44. doi: 10.3390/antib14020044 (PMC12101336; doi:10.3390/antib14020044)
Supplement: Supplementary file 1 [file antibodies-14-00044-s001.zip › Table S2_supplementary file.pdf]

Table S2. Classification of serum samples tested in IgM ELISA based on chimeric recombinant *T. gondii* antigens.

Antigen	Number of tested sera	Classification	Criteria	Reference
EC2 ($MIC2_{157-235}$-$MIC3_{234-307}$-$SAG1_{182-312}$)	157	Acute infection sera (n=50)	IgG^{+}, IgM^{+}	[101]
		Chronic infection sera (n=50)	IgG^{+}, IgM^{-}	
EC3 ($GRA3_{36-134}$-$GRA7_{24-102}$-$M2AP_{37-263}$)		Negative infection sera (n=27)	IgG^{-}, IgM^{-}	
		Serum samples from infants born to mothers with acute toxoplasmosis. (20 congenitally infected and 10 uninfected)		
MAP1 (SAG1- GRA7- GRA1)	250	Acute infection sera (n=25)	IgM^{+} (titer ≥ 64), IgG^{+} (titer ≤1:256) by IFA and HAI, low IgG avidity	[102]
		Persisting IgM (n=25)	IgM+ (titer <64), IgG^{+} (titer ≥ 28) by IFA and HAI, high IgG avidity	
		Chronic infection sera (n=100)	IgM^{-}, IgG^{+} (titer ≥ 256) by IFA and HAI, high IgG avidity	
		Negative infection sera (n=100)	IgG^{-}, IgM^{-}	
MEP ($SAG1_{309-318}$-$SAG2_{109-118}$-$SAG3_{347-356}$)	161	Acute infection sera (n=58)	IgG^{+}, IgM^{+} by ISAGA	[103]
		Chronic infection sera (n=68)	IgG^{+}, IgM^{-} by ISAGA, high IgG avidity	
		Negative infection sera (n=35)	IgG^{-}, IgM^{-}	
P35-MAG1	123	Acute infection sera (n=22)	IgG^{+}, IgM^{+}, low or borderline IgG avidity	[104]
MIC1-ROP1		Chronic infection sera (n=61)	IgG^{+}, IgM^{-}, high IgG avidity	
MAG1-ROP1		Negative infection sera (n=40)	IgG^{-}, IgM^{-}	
$SAG2_{31-170}$-$GRA1_{26-190}$-$ROP1_{85-396}$-$AMA1N_{67-287}$	207	Acute infection sera (n=48)	IgG^{+}, IgM^{+}, low IgG avidity	[105]
$AMA1N_{67-287}$-$SAG2_{31-170}$-$GRA1_{26-190}$-$ROP1_{85-396}$		Persisting IgM (n=18)	IgG^{+}, IgM+, high IgG avidity	
$AMA1C_{287-569}$-$SAG2_{31-170}$-$GRA1_{26-190}$-$ROP1_{85-396}$		Chronic infection sera (n=58)	IgG^{+}, IgM^{-}, high IgG avidity	
$AMA1_{68-569}$-$SAG2_{31-170}$-$GRA1_{26-190}$-$ROP1_{85-396}$		Negative infection sera (n=83)	IgG^{-}, IgM^{-}	
